# Nutritional and Health-Promoting Value of Poultry Meatballs with the Addition of Plant Components

**DOI:** 10.3390/foods11213417

**Published:** 2022-10-28

**Authors:** Anna Augustyńska-Prejsnar, Zofia Sokołowicz, Małgorzata Ormian, Renata Tobiasz-Salach

**Affiliations:** 1 Department of Animal Production and Poultry Products Evaluation, Institute of Food Technology and Nutrition, College of Natural Science, University of Rzeszow, 35-959 Rzeszow, Poland; 2 Department of Crop Production, Faculty of Biology and Agriculture, University of Rzeszow, 35-959 Rzeszow, Poland

**Keywords:** nutritional value, quality, linseed seeds, amaranth seeds, hemp seeds

## Abstract

The aim of the study was to use plant additives in the form of a mixture of seeds (linseed, amaranth and hemp) to increase the nutritional value and health-promoting values of poultry meatballs. Meatballs with 24% addition of wheat roll and three variants of meatballs: M_1_, M_2_, M_3_, with 24% addition seeds were tested. In the meatballs from group M_1_, the percentage of linseeds was 6%, amaranth 10%, and hemp 8%, respectively; in group M_2_ it was 8%, 8%, and 8%; and in group M_3_ it was 10%, 6%, and 8%. When assessing the meatballs quality, the traits taken into account included nutritional value, physical and microbiological traits, and sensory quality. It was found that meatballs enriched with a mixture of seeds were characterized by increased nutritional value (higher content of protein and methionine, polyunsaturated fatty acids, more favorable ratio of n-6 to n-3 acids (3:1), higher content of vitamin E and fiber), brighter color, inferior tenderness compared to meatballs with the addition of wheat roll, and at the same time, desirable sensory traits and acceptable taste. The introduction of a mixture of linseed, amaranth and hemp seeds made it possible to obtain a product targeted at a wide group of consumers, including those on a gluten-free diet.

## 1. Introduction

In recent years, food, including meat products, is no longer seen only as a source of nutrients. Increasingly, attention is paid to the relationship between diet and human health [1,2]. A diet with meat may either have a beneficial effect on human health or constitute the basis for the development of many diet-related diseases, the increasing frequency of which makes it necessary to modify the composition of meat products in order to increase the proportion of bioactive substances with beneficial effects on health [3,4,5]. Consumers of processed meat, aware of the relationship between the diet and human health and the importance of meat in the diet, as well as the dangers of excessive consumption of meat, are increasingly looking for products with high nutritional value and increased pro-health values compared to their current counterparts offered on the market [2,6]. The nutritional value of meat products is mainly determined by the proportion of protein, fat, vitamins and minerals [7]. The pro-health values of meat products can be increased by increasing the share of, among others, fiber, vitamins, essential fatty acids [2,8,9], and the reduction or elimination of synthetic additives [1,2,10]. Enriching meat products with natural bioactive plant compounds or reducing the content of chemical ingredients commonly used in processing are the most commonly used strategies in the production of healthy food [2,6,11]. The use of plant additives in the form of seeds, dried fruits and vegetables, and fruit and vegetable waste in meat processing and their impact on the quality, shelf life, and human health of the final product is currently of great interest to researchers [12,13,14,15,16,17,18,19].

As indicated in the literature [9,11,20,21,22,23,24,25,26,27,28,29,30,31,32], due to the rich nutritional composition of hemp, amaranth, and linseed, they can be used to improve the health-promoting values of meat products. Linseed seeds are a source of many nutrients and bioactive ingredients. They contain about 40% fat, of which almost 60% are Omega-3 fatty acids, and only 15% are Omega-6 fatty acids [20,23]. Fiber accounts for 28% of dry matter, of which about 25% are soluble fractions with properties that reduce the concentration of total and LDL cholesterol. The mineral content is 3–8%. Amino acid composition with a high content of leucine, lysine and phenylalanine of flaxseed has unique biological properties [24]. Phytoestrogens are found in the seeds in the form of sterols (mainly lignans) with antioxidant and anticancer properties [9,25]. The main advantage of amaranth seeds is protein (both its high content and amino acid composition). Amaranth protein contains all essential amino acids, including a relatively large amount of lysine, tryptophan and sulfur amino acids [9,21,26]. The content and composition of lipids are also advantageous, as well as the presence of a valuable component, squalene, which has antioxidant properties and increases the immune resistance of the organism. Dietary fiber has a hypolipemic effect and increases the excretion of bile acids and cholesterol with the feces. Noteworthy is the high content of the minerals iron and calcium [27,28]. Hemp seeds are a rich source of fat (25–35%) and protein. The composition of the fat is beneficial from a nutritional point of view due to the high proportion (75%) of essential fatty acids (EFA) and the favorable ratio of n-6/n-3 acids. Hemp fat also contains large amounts of bioactive compounds, including phytosterols, carotenoids, and polyphenols of anti-carcinogenic and anti-inflammatory nature. Hemp protein is characterized by a good amino acid composition and high digestibility, thanks to which it can be effectively used by the body and is a source of bioactive peptides with antioxidant properties [29,30]. A characteristic feature of hemp seeds is the presence of protein (edestin), phytic acid, choline, trigonellin, lecithin, vitamin K, and tocopherols. Hemp seeds are a good source of vitamin E, iron, calcium, zinc, phosphorus, and magnesium [31,32].

The aim of the study was to use plant additives in the form of a mixture of seeds (linseed, amaranth and hemp) rich in bioactive ingredients to increase the nutritional value and health-promoting values of poultry meatballs. The achievement of this goal was possible thanks to the appropriate composition of the seed mixture and the developing the technology for their grinding and heat treatment.

## 2. Materials and Methods

### 2.1. Poultry Meatballs Ingredients

Poultry meat (thigh muscles of slaughter turkeys, without skin and bones) was purchased from a butcher shop in Rzeszow, Poland and transported in a cooler (4 °C ± 2 °C) to the laboratory. The chemical composition of raw meat was as follows: total ash 1.03%, protein 20.27%, lysine 1.77%, methionine 0.58%, cysteine 0.24%, tryptophan 0.23%, fat 4.18%, and n-6/n-3 fatty acids: 30.76/5.60%.

The plant additives were golden linseed, amaranth and hemp seeds without the husk and were produced by the company Bio Planet, certified as a product from organic farming PL-EKO-07. Dried linseeds with a total ash content of 3.68%, protein 22.51%, lysine 0.87%, methionine 0.42%, cysteine 0.42%, tryptophan 0.36%, fat 35.92%, acids n-6/n-3 fatty acids: 22.90/42.50%. Flaxseed after heat treatment: total ash 2.82%, protein 18.34%, lysine 0.36%, methionine 0.17%, cysteine 0.15%, tryptophan 0.15%, fat 23.48%, n-6/n-3 fatty acids: 21.40/43.20%.

Dried amaranth seeds had a total ash content of 3.41%, protein 15.35%, lysine 0.76%, methionine 0.29%, cysteine 0.29%, tryptophan 0.20%; fat 6.72%, n-6/n-3 fatty acids: 49.00%/10.10%. Amaranth seeds after heat treatment: total ash 2.89%, protein 11.17%, lysine 0.42%, methionine 0.18%, cysteine 0.17%, tryptophan 0.11%; 4.60% fat, n-6/n-3 fatty acids: 43.90/10.20%.

Hemp seeds had a total ash content of 5.17%, protein 32.54%, lysine 1.07%, methionine 0.76%, cysteine 0.51%, tryptophan 0.36%; fat 53.09%, n-6/n-3 fatty acids: 56.90/17.40%. Seeds after heat treatment: total ash 4.20%, protein 26.36%, lysine 0.75%, methionine 0.50%, cysteine 0.30%, tryptophan 0.25%; fat 40.87%, n-6/n-3 fatty acids: 56.20/17.30%.

The wheat roll was purchased from a local bakery. The wheat roll contained ash 2.62%, protein 6.94%, lysine 0.12%, methionine 0.11%, cysteine 0.10%, tryptophan 0.06%, fat 1.09%, n-6/n-3fatty acids: 37.80/4.80%. The hydrated wheat roll contained ash 0.86%, protein 5.78%, lysine 0.10%, methionine 0.08%, cysteine 0.08%, tryptophan 0.05%, fat 0.65%, n-6/n-3: 37.68/4.40%.

The remaining ingredients, such as non-iodized Kłodawskanatural salt, black pepper by the Dary natury company and quail eggs by Bio Planet, were purchased at the health food store Przyprawy bez chemii (spices without chemicals).

### 2.2. Preparation of Fortified Poultry Meatballs

Before the production of poultry meatballs, the meat (20 kg) was cut into cubes of 3–4 cm and cooled to 0 °C ± 2 °C. It was ground twice in a meat grinder (Zelmer, Rzeszów, Poland) with a mesh with a hole diameter of 3 mm, weighed with an accuracy of 1 g and divided into four parts (5 kg each).The linseeds were fragmented (the fragmentation fraction had a particle size of 0.02 ≤ Φ ≤ 2.00 mm), poured with water and heated to a temperature of 90 °C ± 2 °C, which was maintained for 10 min and then cooled down to 20 °C ± 2 °C. Amaranth seeds were soaked in 10 °C ± 2 °C water for 30 min and steamed, maintaining the temperature of 90 ± 2 °C for 10 min and cooled to 20 °C ± 2 °C, and then crushed (the fragmentation fraction with a particle size of 0.02 ≤ Φ ≤ 2.00 mm). Hemp seeds were crushed (the fragmentation fraction with a particle size of 0.02 ≤ Φ ≤ 2.00 mm), then heated to obtain 90 °C ± 2 °C and cooled to 20 °C ± 2 °C.

In each of the two experimental series, four variants of poultry meatballs (WR, M_1_, M_2_, M_3_) with a constant recipe composition were produced (Table 1). The WR group consisted of standard poultry meatballs with the addition of wheat roll, which were treated as the control group. Groups M_1_, M_2_, M_3_ were experimental groups in which, as a substitute for a wheat roll in poultry meatballs, a mixture of linseed, amaranth and hemp seeds in various proportions (Table 1) was added. In each group, all ingredients were put into a mechanical meat mixer with a stainless steel agitator (Titanium, Havand, UK) and mixed for 5 min until the ingredients were evenly distributed. Ball-shaped meatballs with a weight of 40 ± 2 g were formed from the prepared meat masses, and they were cooled in a FKv36/10 refrigerated cabinet (Liebherr, Ulm, Germany) at 4 °C for 0.5 h in order to fix the shape. Before the series of tests was carried out, the poultry meatballs were removed from the cold store and subjected to roasting (at 180 °C until obtaining 80 °C in the geometric center, without moistening). After cooling down, the poultry meatballs were stored in refrigerated conditions (4–6 °C) for 12 h.

### 2.3. Assessment of Physical Traits

The pH of the poultry meatballs was measured using a digital pH meter HI 99163 (Hanna Instrument Company, Woonsocket, RI, USA) equipped with an FC232 electrode (Hanna Instrument Company, Woonsocket, RI, USA) calibrated in pH 4.01 and pH 7.01 buffers (Hanna Instrument Company, Salaj, Romania). The mean pH value was determined from 10 measurements of the same product and the procedures were the same for all samples. The cooking losses were calculated by weighing the samples before and after thermal treatment according to the formula: weight before roasting—weight after roasting/weight before roasting ×100.

The colour test of the cut surface of the meatballs was performed using a colorimeter (CR-300; Minolta Camera, Osaka, Japan). The device was calibrated on a white standard prior to testing. D65 illuminant and a standard colorimetric observer with a field of view of 2° were used for color measurement. Product colour was shown as lightness (L*), redness (a*), and yellowness (b*) in accordance with the International Commission on Illumination (CIE) colour systems. The temperature of the products during all physical measurements was equal to the ambient temperature and amounted to 20 °C ± 2 °C. Product tenderness was evaluated by shear force (Fmax) using a Zwick/Roell machine BT1-FR1 (Zwick, Ulm, Germany), with the Warner-Bratzler type single-blade cutting system (one 1.2 mm flat knife with a triangular indentation of 60°, the inner edge of which is also the working edge). Before cutting, the poultry meatballs were formed into cubes with dimensions of 20 × 20 × 50 mm by cutting off the outer surfaces. The speed of the initial shear force was assumed to be 5 mm/min and 0.2 N, and the time to the initial force being 60 s. After the initial strength was achieved, the actual study was performed at the blade speed of 100 mm·min^−1^ and 0.2 N, until the samples were completely cut (min. three repetitions for each sample). The cutting measurement results were compiled using Test Xpert II software.

### 2.4. Chemical and Microbiological Characteristics

Ash content was determined by burning the samples at 550 °C ± 25 °C in a muffle furnace (Nobetherm P330, Lilienthal, Germany). Protein content (N × 6.25) in raw materials and poultry meatballs was determined by the Kjeldahl method using the titration-distillation method (Kjeldatherm by Gerhardt, Königswinter, Germany, with controlled temperature control, Vapodest Carousel automatic distiller by Gerhardt, Königswinter, Germany). The fat content of raw materials and meatballs was determined by the extraction-weight method (Soxhlet-Soxtherm extraction apparatus by Gerhardt, Königswinter, Germany with an electric dryer enabling the temperature to be maintained in the range of 103 ± 2 °C.

The determination of dietary fiber content in poultry meatballs was performed using the enzymatic-weight method (Kjeldatherm mineralization block by Gerhardt, Königswinter, Germany, Vapodest Carousel automatic distiller by Gerhardt, Germany, vacuum filtration set by Foss Analytical A/S, Denmark). The samples were dried for 12 h and then degreased using petroleum ether, and ground into particles smaller than 0.3 mm. MES/TRIS buffer, c = 0.05 mol/L, was added and adjusted to pH 8.3. They were incubated, adding a solution of α-amylase, protease and amyloglucosidase successively. After the enzymatic decomposition step, the sample was quenched with heated ethyl alcohol (78%) to form a precipitate and filtered under vacuum through a glass filter crucible. The crucible with the sediment was dried for 12 h at (103 ± 2 °C) and then weighed. In one crucible the ash content was determined by calcining in an oven at (550 ± 25 °C), and in the other, nitrogen was determined in the same way as in the protein determination. From the obtained values, the content of dietary fiber in the tested sample was calculated.

Vitamin E (DL-alfa-Tocopherol acetate and Tocopherol) in the products was determined by chromatography REG (EC) [33].

The content of amino acids in raw materials and in poultry meatballs was determined by chromatography acc. to Regulation EC 152/2009 [34]. The percentage of fatty acids in the total fatty acids in raw materials and meatballs was carried out using the method DGF C-VI 11a:2016 mod +DGF C VI 10a: 2016 mod (Agilent Technologies 7890A GC System with FID Detektor and a CP-Sil 88 Säule from the company Agilent, Santa Clara, CA, USA). The principle of the method was based on the separation of fatty acids (identification of fatty acids after the retention time) using the gas chromatography technique with flame ionization detection. Preparation of samples of fatty acid methyl esters-transesterification with BF3 boron trifluoride was performed in accordance with PN-EN ISO 12966-2 [35]. The sample was measured in accordance with PN-EN ISO 12966-4 [36]. Gas chromatography of fatty acid methyl esters was determined by capillary gas chromatography. The transesterified sample solution was spread by a gas chromatograph by flow split injection on a CP-Sil column and analyzed by a flame ionization detector. The acid number was determined by the titration method PN-EN ISO 660:2010 [37].

The microbiological quality assessment (total number of bacteria) of the poultry meatballs was made after 48 h of refrigerated storage of the products (5 °C). With a sterile scalpel, 10 g of the product from each test group was taken and transferred to sterile dishes which were stored in refrigerated conditions at 4 °C. The samples were homogenised in 45 mL of sterile saline (0.9% NaCl) in a BagMixer^®^ laboratory homogenizer (stomacher) and serial dilutions of 10-3 and 10-4 were performed. Cultures were then made on Trypticasein Soy Lab-Agar TSA (Biocorp, Cournon-d’Auvergne, France) and incubated at 37 °C for 24 h to calculate the parameters of the colony-forming units (cfu/g).

### 2.5. Evaluation of the Sensory

The sensory evaluation of poultry meatball samples was carried out by a 10-person evaluation team with proven sensory sensitivity and at least 4 years of experience in carrying out sensory evaluations using the scaling method. A 5-point hedonic scale was used, assessing the intensity of smell and taste, the desirability of smell and taste, juiciness, tenderness, binding, consistency, structure and general desirability, according to the sensory evaluation card [38]. For correct evaluation, the meatball samples were sliced, and the product elements of 2 cm × 2 cm × 3 cm were assigned for evaluation. Sample sets for individual appraisers were coded individually and presented in a given order, which was changed during the second evaluation session in order to avoid the possible influence of the previous sample evaluation on the next sample evaluation. Between each examination of the sample, the panelists took a break (30 s) and rinsed their mouths with mineral water. Each panelist assessed a sample in triplicate. The assessment was carried out in a room free from odors at a temperature of 20 °C ± 2 °C, in accordance with the applicable standard [39].

### 2.6. Statistical Analysis

The obtained data were collated and submitted for statistical analysis using Statistica 13.3. The arithmetic mean and SEM were calculated. The results on the effect of the addition of golden flax, amaranth, and hemp seeds were verified using a one-way analysis of variance. The collected data were checked for normality with the Kolmogorov–Smirnov test with Lilliefors correction. The homogeneity of variances was checked with the Brown–Forsythe test. To indicate the significance of differences between means in groups, we used a Tukey’s test at a 95% confidence level (α = 0.05). Differences were considered as significant if *p* < 0.05. The results on the effect of addition of golden flax amaranth and hemp seeds on sensory properties of pâté were verified with the use of non-parametric Kruskal–Wallis tests.

## 3. Results and Discussion

In the study, there was no effect (*p* > 0.05) of the addition of a seed mixture in any of the studied groups on the pH of poultry meatballs, which ranged from 6.21 to 6.25 (Table 2). Similar results were obtained/published by Bilek and Turhan [23] for beef cutlets with the addition of linseed flour, Kotecka-Majcharzak [30] in pork meatballs with the addition of hemp cake, and Guo et al. [28] in cooked pork with amaranth seed extract.

Significant differences (*p* < 0.05) were found in cooking losses (Table 2). Poultry meatballs in the addition of a mixture of linseed, amaranth and hemp seeds were characterized by lower losses after thermal treatment compared to group WR, with the addition of wheat roll, which was probably due to the retention of moisture in enriched products. Among the study groups, the M_2_ meatballs in which a mixture of seeds of equal proportions (8%) was used, were characterized by lower cooking losses, which may be of technological importance. The reduction of cooking losses in the ground meat product with the addition of hemp ingredients (hemp flour, hemp protein and whole hemp seeds) was demonstrated by Zając et al. [29]. However, the size of the losses was determined not only by the proportion, but also by the form of the additives used. Similar results were obtained by Bilek and Turhan [23], simultaneously showing the reduction of cooking losses with the addition of linseed flour to beef cutlets, and Longato et al. [21] in poultry burgers with amaranth seeds and Sharoba [40] in sausages with amaranth flour.

The color of the product is an important visual feature, influencing the assessment of its quality, determining the choice and decision about a potential purchase or consumption. Additives and their participation in the recipe play an important role in shaping the color of enriched meat products, in addition to the raw meat [29]. The present author’s study showed a significant (*p* < 0.05) effect of the additives used in the mixture of linseed, amaranth and hemp seeds on the color of the cross-section of finished products. Meatballs enriched with seeds, regardless of the proportion of additives used, had a brighter color (higher yellow color brightness L* and saturation b* index and lower color saturation towards red a*). The proportion of meat raw material in all groups was the same, so the color changes of poultry meatballs resulted from the type of plant additives used. Also, Novello et al. [11] noted an increase in the brightness and saturation of the yellow color of beef cutlets with the participation of seeds and linseed flour. However, a decrease in red color intensity a* in beef cutlets with the addition of linseed flour was noted by Bilek and Turhan [23]. The color changes could be due to the presence of yellow flavonoids in linseeds [11]. Kotecka-Majchrzak et al. [30] showed that the colour of the meatballs section changed with a greater share of hemp cake towards darker. Zając et al. [29] showed that the color parameters of pork meatballs changed depending on the hemp ingredient used. The studies by Zając and Świątek [41] showed no effect from the addition of hemp and flax seeds on the quality of pâtés.

The tenderness of the meat product is one of the textural partners of importance to the consumer. The changes in texture parameters depend on the type and amount of the ingredient used and the interaction of the additive with the meat stuffing [29]. The present author’s study showed that poultry meatballs with the addition of a mixture of linseed, amaranth and hemp seeds were characterized by poorer brittleness (greater cutting force) compared to the control group (WR). Among the assessed meatballs enriched with plant additives, the highest hardness was found in group M_3_ of meatballs with the highest proportion of non-husked linseeds. An increase in hardness (increase in sheer force) of poultry burgers enriched with linseed flour and an increase in the hardness of burgers with an increase in the used additive was demonstrated by Cocaro et al. [42]. The increase in hardness measured by the TPA test of meat products enriched with plant additives (hemp and linseed seeds) was confirmed in the study by Zając and Świątek [41], with hemp ingredients by Zając et al. [29], and with amaranth flour by Faid [26] and Tamsen et al. [10].

The indicator of the microbiological quality of food and the health safety of a meat product is the total number of aerobic bacteria [40]. The study showed that products enriched with the addition of linseed, amaranth and hemp seeds were microbiologically safe. In the study groups M_1_ and M_2_ with a higher proportion of amaranth seeds, they inhibited the total number of microorganisms on the third day of refrigerated storage (Table 2). Amaranth seeds contain antimicrobial compounds such as alkaloids, polyphenols, terpenoids and squalene. Confirmation of the antimicrobial effect of amaranth seed extract in cooked pork meat was demonstrated by Guo et al. [28] and of amaranth flour in chicken meat burgers by Longato et al. [21] and in beef sausages by Sharoba 40]. Moreover, Sharoba [40] showed that the total number of aerobic bacteria and the value of the TBA index in the finished product, at the first evaluation date, as well as during storage, decreased with an increase in the proportion of amaranth seed flour. In the study by Zając et al. [29], the addition of hemp ingredients (hemp flour, hemp protein and whole hemp seeds) to pork loaves did not inhibit or increase the total number of microorganisms in relation to the control group and between the product variants.

The addition of a mixture (M_2_) with equal proportions of seeds as a substitute for a wheat roll (WR) caused a significant (*p* ≤ 0.05) increase in the ash content in poultry meatballs (Table 3). In studies by Billek and Turchan 23], Nowello et al. [11], and Cócaro et al., [42] the addition of linseed in the form of seeds or flour increased the ash content in meat products.

Proteins are an essential component of the human diet, and the consumption of the right amount of wholesome protein is important for health [43]. In the present study, the use of a mixture of linseed, amaranth and hemp seeds (groups M_1_, M_2_ and M_3_) as a replacement for wheat roll (WR) had a beneficial effect on increasing the protein content (*p* ≤ 0.05) in poultry meatballs (Table 3). Other authors have also shown similar results. Verma et al. [9] found an increase in the protein content in meatballs with the addition of amaranth; Novello et al. [11] in beef cutlets with the addition of linseed flour, and Novello and Rodrigues Pollonio [20] in patties with the addition of linseed. Poultry proteins contain all of the essential amino acids [44]. In the present study, the amino acid profile of meatballs with the mixture of seeds was better in terms of the content of methionine. Of the nine essential amino acids that cannot be synthesized in the human body (phenylalanine, valine, threonine, tryptophan, methionine, leucine, isoleucine, lysine and histidine) in the amino acid profile of meatballs with a mixture of linseed, amaranth and hemp seeds (M_1_, M_2_ and M_3_), a higher proportion of methionine was found than in meatballs from the control group (WR). The higher methionine content in meatballs with the seed mixture is beneficial and results from the higher methionine content in each of the components of the seed mixtures than in wheat roll. Increasing the proportion of methionine can be considered beneficial for improving the health and nutritional value of poultry meatballs, as methionine has anti-atherosclerotic properties, limiting lipid peroxidation (self-oxidation). In the study by Zeinab et al. [45], the addition of flax seeds to beef sausage did not increase the methionine content in the final product.

In the present study, the modification of the recipe of poultry meatballs resulted in an increase in fat content in all study groups. The fat in enriched meatballs, however, was characterized by a more favorable fatty acid profile than the fat in meatballs with wheat roll (WR). According to Bernacchia et al. [46], linseeds may be a good source of fatty acids in a diet low in marine fish, which is considered the best source of omega-3 fatty acids. The fat of poultry meatballs with seed mixtures compared to the fat of meatballs with wheat roll (WR) contained over 30% less saturated fatty acids (SFA), which significantly improved the health benefits of the meatballs (Table 4). The main ingredient of meatballs is meat, which has a high content of saturated fatty acids [47], considered to be one of the causes of cardiovascular disease. Also, the increase in polyunsaturated fatty acid (PUFA) content in meatballs with the addition of a seed mixture can be considered favorable [48]. In the present study, the proportion of polyunsaturated fatty acids in all study groups was more than 50% higher than in meatballs with wheat roll (WR), which indicates clearly better health benefits of enriched products (Table 4). Among polyunsaturated fatty acids, omega-3 fatty acids play a special role, as in the human body they reduce the concentration of undesirable excess triglycerides in the blood, normalize blood pressure, have anticoagulant and anti-inflammatory effects, inhibit the development of coronary heart disease, support brain function, inhibit excessive immune response, have anti-atherosclerotic properties, support vision processes, and inhibit lipogenesis [49]. Neither omega-3 nor omega-6 acids can be synthesized in the human body due to the lack of enzyme systems capable of creating a double bond in the chain of fatty acids further than C-9 [50]. In the human body, n-3 and n-6 fatty acids are part of the phospholipids of cell membranes, and their mutual proportion in tissues depends to a large extent on the supply in the diet. In our study, the content of n-3 acids beneficial to health was over four times higher (*p* < 0.05) in M_1_ meatballs, five times higher in M_2_ meatballs, and about 4.5 times higher in M_3_ meatballs compared to meatballs from the group WR, which can be associated mainly with the high content of alpha linolenic acid (C18: 3n-3 ALA) in the seeds of linseed contained in the seed mixtures added. Alpha-linolenic acid (18: 3 n-3, ALA) is not synthesized in the human body and must be supplied with the daily diet. The level of omega-3 fatty acids in the blood thus reflects the amount of ALA consumption. In the human body, ALA is converted into eicosapentaenoic acid (20: 5 n-3, EPA) and docosahexaenoic acid (22: 6 n-3, DHA). In light of the cited data, it is worth noting that the tested poultry meatballs with the seed mixture were characterized by a higher proportion of linoleic acid. An increase in the content of n-3 fatty acids after the addition of linseed to ground beef cutlets was also found by Novello et al. [11]. Zając et al. [29] indicate an increase in the content of polyunsaturated fatty acids in a meat product with the addition of hemp seeds.

One of the indicators of the quality of a healthy human diet is not only the level of consumption of omega-3 PUFAs, but also the proportion of omega-6 fatty acids to omega-3 fatty acids. Although the content of both omega-3 and omega-6 fatty acids in the diet is important, an excess of omega-6 fatty acids may interfere with the enzymes responsible for the desaturation and elongation of omega-6 and omega-3 fatty acids and hinder the conversion of ALA to EPA and DHA [50,51]. In addition, the ratio of omega-6 to omega-3 in the diet affects the functioning of neurotransmitters and the brain. A diet containing excessive amounts of polyunsaturated fatty acids (PUFA) and a high ratio of omega n-6 to omega n-3, increases the susceptibility to cardiovascular diseases, cancer, inflammatory and autoimmune diseases, and an increased level of omega-3 PUFA (low omega-6/omega 3) reduces the risk of these diseases [52,53]. In the present study, the ratio of omegan-6/omega n-3 fatty acids in poultry meatballs with wheat roll (WR) was not favorable and amounted to 10:1, while in meatballs with a mixture of hemp, amaranth and golden linseed seeds it was favorably narrowed to 3:1 in groups M_1_ and M_3_ and 2:1 in group M_2_, respectively, which is the level considered optimal in the human diet. An improvement in the ratio of omega n-6 to omega n-3 was achieved by Bilek and Turchan [23] in beef cutlets with linseed flour; Bilska et al. [54] and Zając and Świątek [41] in pâtés with 20% addition of linseed oil and hemp and linseed seeds, and Zeinab et al. [45] in beef sausage with the addition of linseeds.

The addition of a mixture of seeds (M_1_, M_2_, M_3_) instead of a hydrated roll (WR) did not increase the share (*p* > 0.05) of trans fatty acids, which are unfavorable to human health, in the finished product. Paying attention to the level of trans fatty acids in poultry meatballs with a mixture of linseed, amaranth and hemp seeds is due to the fact that unsaturated trans fatty acids incorporated into cell membranes, instead of cis isomers, cause changes in membrane permeability, activity and the number of receptors and enzymes associated with membranes, which is associated with the deterioration of the vital functions of these cells. Trans fatty acids have been shown to have a significant impact on the risk of developing atherosclerotic lesions and cardiovascular disease [55].

Fiber is considered a dietary component with a beneficial effect on health and is widely used in modern technologies to improve the functional values of meat products [3,9]. Dietary fiber has a beneficial effect on human health as a factor in preventing many diseases, including cancers of the gastrointestinal tract. It contributes, among other things, to reducing the absorption of cholesterol and triglycerides, lowering blood pressure, slowing down the breakdown of carbohydrates, and lowering blood glucose levels. Fiber accelerates intestinal peristalsis and the passage of contents through the digestive tract, prevents constipation, promotes the development of beneficial intestinal bacteria, and reduces the feeling of hunger [56]. In our study, the addition of a mixture of seeds caused a significant (*p* ≤ 0.05) increase in the proportion of fiber in poultry meatballs from groups M_1_, M_2_, and M_3_. The possibility of using hemp ingredients in meat processing also drew the attention of Zając et al. [29], who, after adding hemp flour, hemp protein, husked and whole hemp seeds, obtained pork containing fiber. In the study by Verm et al. [9], gluten -free, fiber-rich lumps of goat meat were obtained using amaranth flour (1.5%) and quinoa flour (3%) as a substitute for wheat flour. Zeinab et al. [45] obtained beef sausage with fiber through the addition of linseed and chickpeas.

Among the vitamins found in meat products, vitamin E deserves special attention, which is considered the most important antioxidant soluble in fat, protecting the human body against various pathological processes related to oxidative stress [57]. Oxidative stress is one of the main factors influencing the development of atherosclerosis and its complications, and atherosclerosis is the source of most cardiovascular diseases. Natural vitamin E consists of a family of eight different compounds of tocopherols and tocotrienols, which are powerful antioxidants that trap lipoperoxyl radicals. Moreover, α-tocopherol also has anti-inflammatory and antithrombotic effects [58,59]. The study showed that enriched poultry meatballs from group M_3_ were characterized by twice as much (*p* ≤ 0.05), and from groups M_1_ and M_2_—almost three times as much vitamin E as compared to the control product (WR). From a nutritional point of view, replacing wheat roll (WR) with a mixture of linseed, amaranth and hemp seeds can be considered as beneficial for increasing the health benefits of poultry meatballs. There is little research work on the use of plant additives to increase vitamin E content in poultry meat products. In the study by Dominguez et al. [60], the increase in the content of α-tocopherol in the meat product was obtained by replacing fat with olive oil.

Sensory attributes are the main determinant of the quality of meat products and their acceptability by consumers. In the production of fortified food, it is important that enriched meat products do not differ from traditional recipe products in terms of sensory characteristics [61]. In our study, the use of the addition of a seed mixture in groups M_1_, M_2_, and M_3_, regardless of the proportion of individual components, had a favorable (*p* < 0.05) effect on smell and taste desirability, as well as the structure and bonding of the products. However, within the research groups, the M_2_ meatballs enriched with 8% of plant additives were rated the best in terms of taste desirability, product binding and general desirability. On the other hand, the tenderness of the enriched meatballs in all study groups was rated lower compared to the product with the addition of wheat roll (Table 5). In the study, the increase in the hardness of the samples in the study groups was probably caused by the use of plant additives with a high fibre content. Fibre, thanks to its characteristic structure and ability to cross-link, allows for strengthening the texture of products with its addition. In the case of meat products made from ground mass, the higher hardness of products does not always reduce their quality, and the consumer expects good bonding and proper structure from such a product [9]. Furthermore, in the studies by Zając et al. [41], the pâté with both hemp seed and linseed and Zając et al. [29] of pork loaves with the addition of hemp seeds, de-hulled hemp seeds, hemp protein and hemp flour was harder than the control product. In the study by Zając and Świątek [41], the overall sensory quality of pâtés with the addition of hemp seeds and golden flax was rated higher compared to the control pâté. Naumova et al. [8] indicate that the 10% addition of hemp flour contributed to the improvement of the taste and aromatic properties of beef cutlets with acceptable consistency and juiciness characteristics; however, the higher proportion of hemp added decreased the sensory characteristics. On the other hand, Kotecka-Majchrzak [30] reported that 10% of hemp cake added causes a significant change in the color and taste of pork meatballs, but it retained acceptable taste characteristics. In studies by Bilek and Turhan [23] it was noted that the addition of linseed flour obtained from linseed seeds had an impact on the assessment of sensory characteristics (appearance, tenderness, juiciness and general acceptability). Verma et al. [9] indicate that the addition of amaranth seeds within the range of 1.5% increased the acceptability of all sensory characteristics of goat meatballs. The results of the study carried out by Sabzi Belekhkanlu et al. [61] showed that the use of amaranth flour in the production of typical meat hamburgers led to a product with good sensory properties acceptable to consumers. On the other hand, Longato et al. [21] showed that amaranth and pumpkin seeds used in the production of poultry burgers did not have a significant effect on sensory characteristics; only burgers with 2% amaranth seeds were rated higher for juiciness and overall acceptability compared to the control burgers. In the study by Zając et al. [29], the overall acceptability of pâtés with the addition of hemp ingredients (hemp flour, hemp protein and whole hemp seeds) was lower for fortified products, and only the taste of the product with the addition of husked hemp seeds was comparable to the control product. Novelo et al. [11] indicate that the addition of 10% golden linseed resulted in a reduction in flavor and texture.

The use of the author’s original mixture of linseed, amaranth and hemp seeds instead of wheat roll for the production of poultry meatballs allowed for obtaining a gluten-free product. Confirmation of the use of amaranth seeds in meat products for people with celiac disease was found in the studies by Tamsen et al. [10], Kerimoglu and Seredaroglu [62].

## 4. Conclusions

The study results indicate that the use of a mixture of linseed, amaranth and hemp seeds as a substitute for wheat roll in poultry meatballs is a technological alternative, allowing for obtaining a product without the participation of synthetic additives, with increased nutritional value, microbiologically safe, and with acceptable physical and sensory properties.

In all study groups, the addition of the seed mixture caused an increase in the protein content, methionine, fiber, vitamin E, n-3 and n-6 fatty acids, which allowed meatballs with an increased health-promoting value. Moreover, the ratio of n6/n3 acids was advantageously narrowed from 10:1 in group WR to 3:1 in groups M_1_ and M_3_ and 2:1 in group M_2_ to the level considered optimal in the human diet. Meatballs enriched with a mixture of seeds were characterized by a brighter color and worse tenderness compared to meatballs with the addition of wheat roll. The beneficial effect of the seed mixture on the desirable smell and taste as well as the structure and binding of enriched meatballs was demonstrated.

Among the study groups, enriched meatballs from group M_2_, in which a mixture of seeds was used in equal proportions, were characterized by lower thermal losses and more favorable taste, product binding and general desirability. The meatballs from group M_3_ with the highest proportion of linseeds were characterized by the greatest hardness.

The obtained meatballs with a mixture of linseed, amaranth and hemp seeds are characterized by high nutritional value and pro-health values and can be intended for a wide group of consumers of meat products, including people on a gluten-free diet.

## 5. Patents

Patent application marked with the number: P.439001 [WIPO ST 10/C PL439001].

## Figures and Tables

**Table 1:** Recipe composition of poultry meatballs (%).

Ingredients		Variants of Product			
		WR	M_1_	M_2_	M_3_
Meat from the thighs of slaughter turkeys		69.80	69.80	69.80	69.80
Seed mixture	linseed	-	6.00	8.00	10.00
	amaranth	-	10.00	8.00	6.00
	husked hemp	-	8.00	8.00	8.00
Wheat roll		24.00	-	-	-
Quail egg mass		5.00	5.00	5.00	5.00
Non-iodized salt		1.00	1.00	1.00	1.00
Black pepper		0.20	0.20	0.20	0.20

**Table 2:** Effect of adding a mixture of linseed, amaranth and hemp seeds on the physical characteristics, total number of bacteria and acid number of poultry meatballs ($\bar{x}\;$± s).

Studied Parameters	Poultry Meatballs				
	WR	M_1_	M_2_	M_3_	*p* Value
pH	6.21 ± 0.01	6.24 ± 0.02	6.25 ± 0.03	6.24 ± 0.02	0.1140
Cooking losses (%)	18.72 ^a^ ± 1.80	15.00 ^b^ ± 2.30	13.01 ^c^ ± 1.50	16.79 ^b^ ± 1.10	0.0107
Colour cross-section:					
L*—lightness	63.12 ^b^ ± 5.08	68.51 ^a^ ± 3.15	66.60 ^a^ ± 5.45	66.34 ^a^ ± 4.29	0.0041
a*—redness	9.09 ^a^ ± 0.84	6.46 ^b^ ± 0.57	6.06 ^b^ ± 0.47	6.61 ^b^ ± 0.56	0.0000
b*—yellowness	13.62 ^b^ ± 1.28	15.06 ^a^ ± 0.65	15.56 ^a^ ± 1.26	15.73 ^a^ ± 0.95	0.0010
Warner–Bratzler shear force (N)	10.94 ^c^ ± 1.10	14.15 ^b^ ± 2.81	13.63 ^b^ ± 4.51	16.97 ^a^ ± 2.81	0.0012
TVCs total viable counts (log cfu/g)	3.19 ^a^ ± 0.29	2.66 ^b^ ± 0.54	2.42 ^b^± 0.42	3.10 ^a^ ± 0.30	0.0000
Acid number (mgKOH/g)	1.10 ± 0.32	0.99 ± 0.12	0.97 ± 0.05	1.04 ± 0.10	0.3201

Explanation: WR—addition of wheat roll 24%, M_1_—proportion of seeds in the product: linseed 6%, amaranth 10%, hemp 8%; M_2_ —linseed 8%, amaranth 8%, hemp 8%, M_3_—linseed 10%, amaranth 6%, hemp 8%; a, b, c—medium values marked with different letters in lines differ statistically significantly.

**Table 3:** Effect of adding a mixture of linseed, amaranth and hemp seeds on the chemical composition of poultry meatballs ($\bar{x}\;$± s).

Studied Parameters	Poultry Meatballs				
	WR	M_1_	M_2_	M_3_	*p* Value
Dry weight (%)	33.55 ^b^ ± 0.21	37.00 ^a^ ± 0.50	36.30 ^a^ ± 0.68	35.15 ^a^ ± 0.96	0.0000
Total ash (%)	1.83 ^b^ ± 0.11	1.99 ^ab^ ± 0.15	2.19 ^a^ ± 0.25	2.01 ^ab^ ± 0.16	0.0239
Protein (%)	18.87 ^b^ ± 0.20	20.39 ^a^ ± 0.24	20.65 ^a^ ± 0.14	20.58 ^a^ ± 1.31	0.0001
Fat (%)	6.55 ^b^ ± 0.49	10.93 ^a^ ± 0.82	11.30 ^a^ ± 1.10	10.91 ^a^ ± 0.62	0.0000
Fiber (%)	<1.00 ^b^ ± 0.00	2.48 ^a^ ± 0.20	2.51 ^a^ ± 0.10	2.18 ^a^ ± 0.40	0.0160
Tocopherol content (mg/kg):	2.96 ^c^ ± 0.83	7.31 ^a^ ± 0.92	7.13 ^a^ ± 1.20	6.36 ^b^ ± 1.68	0.0000
Vitamin E, as DL-alfa-Tocopherol acetate (mg/kg)	2.69 ^c^ ± 0.75	6.65 ^a^ ± 0.86	6.52 ^a^ ± 0.92	5.79 ^b^ ± 1.53	0.0000
Amino acid content (%):					
Lysine	1.50 ± 0.13	1.57 ± 0.10	1.53 ± 0.20	1.59 ± 0.07	0.1364
Methionine, expressed as methionine sulfone	0.46 ^b^ ± 0.06	0.52 ^a^ ± 0.10	0.52 ^a^ ± 0.12	0.56 ^a^ ± 0.05	0.0001
Cysteine, expressed as cysteic acid	0.21 ± 0.04	0.25 ± 0.08	0.25 ± 0.04	0.25 ± 0.01	0.1212
Aspartic acid	1.64 ^b^ ± 0.14	1.83 ^a^ ± 0.16	1.80 ^a^ ± 0.20	1.84 ^a^ ± 0.06	0.0001
Threonine	0.80 ± 0.07	0.85 ± 0.04	0.84 ± 0.05	0.86 ± 0.03	0.2111
Serine	0.76 ± 0.06	0.83 ± 0.08	0.80 ± 0.04	0.82 ± 0.03	0.1625
Glutamic acid	3.06 ± 0.20	3.11 ± 0.12	3.01 ± 0.12	3.12 ± 0.12	0.1124
Proline	0.82 ± 0.05	0.73 ± 0.08	0.71 ± 0.04	0.74 ± 0.03	0.3082
Glycine	0.89 ± 0.11	0.91 ± 0.10	0.88 ± 0.08	0.92 ± 0.03	0.3944
Alanine	1.04 ± 0.09	1.06 ± 0.04	1.04 ± 0.05	1.07 ± 0.04	0.0584
Valine	0.90 ± 0.07	0.97 ± 0.04	0.94 ± 0.04	1.07 ± 0.03	0.2232
Isoleucine	0.86 ± 0.06	0.91 ± 0.06	0.89 ± 0.04	0.92 ± 0.03	0.0621
Leucine	1.44 ± 0.09	1.50 ± 0.05	1.47 ± 0.04	1.51 ± 0.06	0.1588
Tyrosine	0.59 ± 0.06	0.63 ± 0.06	0.61 ± 0.04	0.63 ± 0.02	0.1152
Phenylalanine	0.76 ± 0.06	0.81 ± 0.02	0.79 ± 0.05	0.83 ± 0.03	0.2141
Histidine	0.50 ± 0.04	0.54 ± 0.01	0.51 ± 0.03	0.53 ± 0.02	0.4112
Arginine	1.16 ^b^ ± 0.09	1.38 ^a^ ± 0.05	1.32 ^a^ ± 0.02	1.37 ^a^ ± 0.03	0.0000
Tryptophan	0.22 ± 0.01	0.23 ± 0.03	0.22 ± 0.03	0.23 ± 0.01	0.4112

Explanation: WR—addition of wheat roll 24%, M_1_—proportion of seeds in the product: linseed 6%, amaranth 10%, hemp 8%; M_2_—linseed 8%, amaranth 8%, hemp 8%, M_3_—linseed 10%, amaranth 6%, hemp 8%; a, b, c—medium values marked with different letters in lines differ statistically significantly.

**Table 4:** Effect of adding a mixture of linseed, amaranth and hemp seeds on the fatty acid profile of poultry meatballs (%).

Studied Parameters	Poultry Meatballs				
	WR	M_1_	M_2_	M_3_	*p* Value
Lauric acid C 12:0	0.30 ± 0.04	0.20 ± 0.03	0.20 ± 0.05	0.20 ± 0.04	0.1421
Tetradecanoic acid C 14:0	1.00 ^a^ ± 0.05	0.60 ^b^ ± 0.03	0.50 ^b^ ± 0.04	0.55 ^b^ ± 0.05	0.0000
Oleomyristic acid C 14:1	0.15 ± 0.07	<0.1 ± 0.00	0.10 ± 0.04	0.10 ± 0.03	0.5724
Pentadecanoic acid C 15:0	0.15 ± 0.07	0.10 ± 0.04	0.10 ± 0.04	0.10 ± 0.04	0.6214
Hexadecanoic acid C 16:0	22.20 ^a^ ± 0.14	15.20 ^b^ ± 0.15	14.40 ^b^ ± 0.28	14.70 ^ab^ ± 0.38	0.0000
Hexadecenoic acid C 16:1	2.95 ± 0.07	2.00 ± 0.09	1.70 ± 0.05	1.65 ± 0.08	0.0625
Heptadecanoic acid C 17:0	0.30 ^a^ ± 0.01	0.10 ^c^ ± 0.01	0.20 ^b^ ± 0.01	0.20 ^b^ ± 0.05	0.0000
Octadecenoic acid C 18:0	8.80 ^a^ ± 0.20	6.10 ^b^ ± 0.15	6.20 ^b^ ± 0.08	6.35 ^b^ ± 0.13	0.0000
Trans-9-octadecanoic acid C 18:1	0.20 ± 0.02	0.20 ± 0.01	0.20 ± 0.01	0.20 ± 0.04	0.9514
Cis-9-octadecenoic acid C 18:1	27.35 ^a^ ± 0.30	21.30 ^b^ ± 0.40	21.00 ^b^ ± 0.42	20.75 ^b^ ± 0.31	0.0000
Cis-11-octadecenoic acid C 18:1	1.95 ^a^ ± 0.07	1.30 ^b^ ± 0.10	1.30 ^b^ ± 0.20	1.40 ^b^ ± 0.05	0.0000
Linoleic acid C 18:2 n-6	28.20 ^b^ ± 0.14	37.30 ^a^ ± 0.14	35.40 ^a^ ± 0.14	37.05 ^a^ ± 0.94	0.0001
Alpha-linolenic acid C 18:3 n-3	2.60 ^c^ ± 0.10	11.60 ^b^ ± 0.25	15.00 ^a^ ± 0.20	13.23 ^a^ ± 1.61	0.0000
Gamma-linolenic acid C 18:3 n-6	<0.1 ^c^ ± 0.00	0.60 ^a^ ± 0.15	0.40 ^b^ ± 0.05	0.49 ^b^ ± 0.08	0.0000
Stearidonic acid C 18:4 n-3	<0.1 ± 0.00	0.20 ± 0.04	0.20 ± 0.02	0.19 ± 0.04	0.3419
Eicosenoic acid C 20:0	<0.1 ^b^ ± 0.00	0.30 ^a^ ± 0.05	0.30 ^a^ ± 0.04	0.30 ^a^ ± 0.02	0.0003
Eicosenoic acid C 20:1	0.40 ± 0.01	0.40 ± 0.03	0.30 ± 0.04	0.34 ± 0.05	0.1264
Eicosadienoic acid C 20:2 n-6	0.30 ± 0.01	0.20 ± 0.01	0.20 ± 0.01	0.20 ± 0.01	0.2145
Arachidonic acid C 20:4 n-6	1.65 ^a^ ± 0.07	1.10 ^b^ ± 0.05	1.10 ^b^ ± 0.04	1.08 ^b^ ± 0.05	0.0441
Docosenoic acid C 22:0	<0.1 ± 0.00	0.10 ± 0.02	0.10 ± 0.03	0.11 ± 0.04	0.0791
Docosatetraenoic acid C 22:4 n-3	0.30 ± 0.01	0.20 ± 0.04	0.20 ± 0.03	0.20 ± 0.02	0.0592
Docosapentaenoic acid C 22:5 n-3	0.20 ± 0.01	0.10 ± 0.05	0.10 ± 0.03	0.14 ± 0.05	0.0731
Docosapentaenoic acid C 22:5 n-6	0.10 ± 0.00	<0.1 ± 0.00	<0.1 ± 0.00	<0.1 ± 0.00	0.0864
Docosahexaenoic acid C 22:6 n-3	0.20 ± 0.01	0.10 ± 0.00	0.10 ± 0.00	0.10 ± 0.00	0.0786
ΣSFA	32.75 ^a^ ± 0.21	22.60 ^b^ ± 0.52	21.90 ^b^ ± 0.40	22.31 ^b^ ± 0.37	0.0000
ΣMUFA	32.80 ^a^ ± 0.42	25.10 ^b^ ± 0.40	24.40 ^b^ ± 0.80	24.59 ^b^ ± 0.42	0.0000
ΣPUFA	33.75 ^b^ ± 0.35	51.80 ^a^ ± 0.85	52.80 ^a^ ± 0.90	52.26 ^a^ ± 0.69	0.0000
ΣTRANS	0.25 ± 0.07	0.20 ± 0.05	0.20 ± 0.05	0.21 ± 0.06	0.0764
Σn-3	3.00 ^c^ ± 0.50	12.10 ^b^ ± 1.20	15.40 ^a^ ± 1.50	13.65 ^b^ ± 1.57	0.0000
Σn-6	30.70 ^c^ ± 0.28	39.40 ^a^ ± 0.60	37.30 ^b^ ± 0.30	39.00 ^a^ ± 0.30	0.0000

Explanation: WR—addition of wheat roll 24%, M_1_—proportion of seeds in the product: linseed 6%, amaranth 10%, hemp 8%; M_2_—linseed 8%, amaranth 8%, hemp 8%, M_3_—linseed 10%, amaranth 6%, hemp 8%; a, b, c—medium values marked with different letters in lines differ statistically significantly.

**Table 5:** Effect of adding a mixture of linseed, amaranth and hemp seeds on the sensory traits of poultry meatballs (points).

Studied Parameters	Poultry Meatballs				
	WR	M_1_	M_2_	M_3_	*p* Value
Smell intensity	4.00 ± 0.46	4.02 ± 0.40	4.10 ± 0.30	4.15 ± 0.25	0.8347
Taste intensity	4.00 ± 0.56	4.20 ± 0.49	4.16 ± 0.49	4.18 ± 0.49	0.7964
Smell desirability	3.78 ^b^ ± 0.62	4.70 ^a^ ± 0.53	4.64 ^a^ ± 0.53	4.68 ^a^ ± 0.53	0.0002
Taste desirability	3.83 ^c^ ± 0.43	4.20 ^b^ ± 0.49	4.88 ^a^ ± 0.49	4.12 ^b^ ± 0.49	0.0001
Juiciness	4.23 ± 0.53	4.00 ± 0.66	4.18 ± 0.66	4.00 ± 0.66	0.8251
Tenderness	4.28 ^a^ ± 0.62	3.60 ^b^ ± 0.56	3.87 ^b^ ± 0.56	3.78 ^b^ ± 0.56	0.0002
Binding	3.67 ^c^ ± 0.43	4.18 ^b^ ± 0.53	4.83 ^a^ ± 0.53	4.10 ^b^ ± 0.53	0.0130
Consistency	4.00 ± 0.53	3.56 ± 0.46	4.10 ± 0.46	3.86 ± 0.46	0.1261
Structure	3.72 ^b^ ± 0.44	4.20 ^a^ ± 0.53	4.54 ^a^ ± 0.53	4.28 ^a^ ± 0.53	0.0271
General desirability	3.96 ^b^ ± 0.57	4.04 ^b^ ± 0.55	4.40 ^a^ ± 0.55	4.05 ^b^ ± 0.55	0.0325

Explanation: WR—addition of wheat roll 24%, M_1_—proportion of seeds in the product: linseed 6%, amaranth 10%, hemp 8%; M_2_—linseed 8%, amaranth 8%, hemp 8%, M_3_—linseed 10%, amaranth 6%, hemp 8%; a, b, c—medium values marked with different letters in lines differ statistically significantly.

## Data Availability

Data are available from the corresponding author.
